# Application of Ketamine in Pain Management and the Underlying Mechanism

**DOI:** 10.1155/2023/1928969

**Published:** 2023-08-16

**Authors:** Xiaofan Ma, Jia Yan, Hong Jiang

**Affiliations:** Department of Anesthesiology, Shanghai Ninth People's Hospital Affiliated to Shanghai Jiao Tong University School of Medicine, Shanghai, China

## Abstract

Since ketamine was approved by the FDA as an intravenous anesthetic, it has been in clinical use for more than 50 years. Apart from its anesthetic effects, ketamine is one of the few intravenous anesthetics with potent analgesic properties. As part of the effort to develop pain management, renewed interest has focused on the use of ketamine for the treatment of acute and chronic pain. Ketamine is commonly used to treat various kinds of chronic pain syndromes and is also applied to control perioperative pain and reduce the consumption of postoperative analgesics. However, its precise mechanisms of action remain mysterious for a large part. Despite extensive research in the field, the mechanism of ketamine is still unclear. Its analgesic effect appears to be largely mediated by blockade of NMDARs, but opioid, GABA, and monoaminergic system seem to partly participate in the pain transmission procedure. Its metabolites also have an analgesic effect, which may prolong pain relief. More recently, the antidepressant effect of ketamine has been considered to reduce pain-related aversion to relieve chronic pain. Overall, the analgesic mechanism of ketamine seems to be a complex combination of multiple factors. Due to its potent analgesic properties, ketamine is an analgesic with great clinical application prospects. Exploring the precise mechanism of action of ketamine will help guide clinical medication and confirm indications for ketamine analgesia. This review aims to list the application of ketamine in pain management and discuss its analgesic mechanism.

## 1. Introduction

Ketamine is a well-established anesthetic drug that has been used clinically for about 50 years [1]. It is a noncompetitive antagonist of the N-methyl-D-aspartic acid receptors (NMDARs) and was first synthesized in 1962 for the relief of the serious psychotomimetic side effects of phencyclidine (PCP) but to retain its anesthetic effects [2, 3]. Ketamine can produce a unique type of anesthesia state called “dissociative anesthesia” where patients appear to be awake but do not respond to apparent surgical stimuli [3]. During anesthesia, ketamine can maintain the airway tone and respiration and increase sympathetic activity, which leads it to be the anesthetic drug of choice in areas with limited resuscitation equipment [4, 5]. In addition, ketamine has a potent analgesic effect with only subanesthetic doses so that it has garnered increasing attention over recent years in pain management [6]. Recently, there has been an increasing use of ketamine to provide an analgesic effect in various kinds of pain, including perioperative pain and chronic pain syndrome [6–9]. However, ketamine's analgesic mechanism of action is still unclear. It may be a complex combination of multiple mechanisms related to blockade of different kinds of molecular receptors, activation of descending inhibitory pathways, relief of the affective-motivational dimensions of pain, and so on that finally results in the analgesic effect [1, 9–11]. In this review, we will briefly summarize the recent clinical findings of ketamine in pain management and discuss the known mechanisms of action from various aspects.

## 2. Methods

In October 2022, we searched PubMed and Web of Science. We searched the mentioned databases using search terms including “ketamine” AND “pain,” “ketamine” AND “analgesia,” “ketamine” AND “analgesia” AND “mechanism,” “ketamine” AND “metabolites,” “ketamine” AND “psychotomimetic,” “ketamine” AND “autobiographical memory.” The reference lists for included studies were manually screened by members to minimize the omission of potentially eligible articles.

## 3. Pharmacological Effects of Different Doses of Ketamine

In high doses, ketamine produces anesthesia and analgesia and is often used in diagnostic and surgical procedures that do not require skeletal muscle relaxation. An average initial dose of 2 mg/kg ketamine can produce 5 to 10 min of surgical anesthesia within 30 s, and an addition of 50% to 100% of induction dose at a rate of 0.1 to 0.5 mg/min will maintain general anesthesia in adult patients [12]. Ketamine is also commonly used as a supplement to other anesthetic drugs. Due to drug interactions, the combination of ketamine with other anesthetic drugs, including halogenated general anesthetics, benzodiazepines, and propofol, can prolong the duration of action and reduce the dose required. Unlike many other intravenous anesthetics, ketamine can maintain the heart rate and cardiac output and increase the blood pressure via sympathetic stimulation [13]. Ketamine can stimulate noradrenergic neurons to release norepinephrine, dopamine, and serotonin and inhibit neuronal catecholamine uptake [14]. In addition to the cardiovascular effects, ketamine provides bronchodilation and preserves protective pharyngeal and laryngeal reflexes without depressing respiration [13]. Its good safety profile makes it particularly suitable for anesthesia and sedation in areas with limited medical equipment.

However, patients often reported a series of psychotomimetic symptoms when recovering from ketamine-induced anesthesia, including delusions, hallucinations, delirium and confusion, and out-of-body or near-death experiences, which has largely hindered the clinical application of ketamine [4]. Research has shown that NMDAR antagonism appears to be responsible for the psychotomimetic effects. S-ketamine, but not R-ketamine, produced behavioral abnormalities in rodents suggestive of psychotic symptoms, such as hyperactivity and prepulse inhibition deficits [15]. Consistently, R-ketamine, which has a lower affinity for the NMDARs than S-ketamine, induced a state of relaxation in healthy volunteers while equimolar doses of S-ketamine produce psychotic symptoms [16]. Based on these findings, we infer that reducing the blockade of NMDARs may alleviate the psychotomimetic side effects of ketamine.

In low doses, ketamine acts as an analgesic drug. The fact that a single injection of 0.15 mg/kg ketamine is sufficient to relieve both acute and chronic pain suggests that ketamine also has an important role in pain management [17, 18], which we detail in Section 4. Moreover, research has demonstrated a linear relationship between the psychotomimetic effects of ketamine and plasma concentrations of 50 to 200 ng/ml, a plasma concentration range that is clinically relevant in patients receiving ketamine for analgesia or sedation, or recovering from ketamine anesthesia [19], which means subanesthetic doses of ketamine are effective in managing acute pain, while having fewer psychotomimetic side effects compared to anesthetic doses.

Furthermore, ketamine has been found to have properties that promote inflammatory homeostasis and act as an immunomodulator [20]. In both animal experiments and clinical trials, ketamine can prevent the general anti-proinflammatory mechanisms, thus avoiding excessive suppression of the pro-inflammatory influences. As a result, ketamine specifically demonstrates the ability to inhibit the escalation and spread of local inflammation, while still allowing for the natural progression and resolution of inflammatory processes [21]. In a systematic review of clinical studies, the authors concluded that intraoperative ketamine significantly inhibited the early postoperative inflammatory response [22]. Therefore, ketamine can not only provide analgesic effects up to six months after surgery but also facilitate rehabilitation at 1 month after total hip arthroplasty [23]. However, these studies were based on inflammatory markers, and the anti-inflammatory effects of ketamine in major surgery should be explored from multiple perspectives. It is important to determine whether ketamine can improve patient prognosis, elucidate the mechanisms underlying its anti-inflammatory effects, and propose an appropriate dosage regimen.

## 4. Clinical Application in Pain Management

In the following sections, various applications of ketamine in pain management are described. A summary of example clinical trials is presented in Table 1.

### 4.1. Role of Ketamine in Perioperative Pain Management

Subanesthetic ketamine, as an adjunct to general anesthesia, reduces postoperative pain and the need for opioids in a variety of situations, including outpatient surgery [24], abdominal procedure [17, 25], orthopedic surgery [23, 26], and spine surgery [27, 34]. In the above study, the main dosing schedule can be summarized as follows: a single intravenous injection of ketamine was used before or after induction of general anesthesia, but before incision, which might be followed by a continuous infusion until the end of the operation or several days after surgery. In 2018, Schwenk et al. published consensus guidelines on the use of intravenous ketamine for acute pain management. They stated that subanesthetic ketamine application should be considered as an adjunct in surgeries in which the expected postoperative pain will be severe and might be used for opioid-dependent or opioid-tolerant patients undergoing surgery [35]. Moreover, the addition of ketamine to a local anesthetic or other analgesics in peripheral or intravertebral anesthesia could improve pain relief and prolong the duration of postoperative analgesia [28–30, 36, 37]. Interestingly, intravertebral administration of the same dose of ketamine provided long-lasting postoperative analgesia and less postoperative analgesic consumption than the intravenous injection of ketamine in both adults [38] and children [39]. Although the dosing schedule of ketamine is adjusted by a variety of factors, such as the type of anesthesia and route of administration, ketamine still seems to be a good adjunct to relieve perioperative pain. The prevailing explanation proposes that these effects may relate to central sensitization, which can be prevented by blockade of NMDARs [7], and we will further discuss this mechanism in Section 5.1.

### 4.2. Role of Ketamine in Prehospital Trauma Care

In addition to relieving perioperative acute pain, ketamine has also been shown to be effective in prehospital trauma care. According to a field report from a medical aid to an earthquake in Italy in 2016, the use of ketamine alone or in combination with other analgesics was effective in reducing patients' pain scores [40]. Besides its analgesic effect, ketamine is preferable as a procedural sedation agent in patients with or at risk for hemorrhagic shock or respiratory distress [41]. At the same time, ketamine is less likely to decrease blood pressure or depress the respiratory system compared with morphine due to its sympathetic exciting effects, suggesting it may be given to trauma patients who are still in pain after receiving opioids or when re-dosing of opioids may be dangerous [41].

It is worth noting that the exposure to a traumatic event such as natural disaster, warfare, traffic collisions, or other threats on a person's life might cause trauma and stress-related disorders such as acute stress disorder (ASD) and posttraumatic stress disorder (PTSD). The characteristic symptoms manifest as flashbacks or intrusive thoughts about the traumatic event. Research has shown that autobiographical memory deficits referred to as overgeneral autobiographical memory (OGM) are likely to play an important role in the trauma-related mental disorders [42]. Autobiographical memory is defined as encompassing both the recollection of personal past events and factual knowledge about oneself [43], consisting of episodic and semantic memories [44]. Interestingly, a single injection of ketamine at a subanesthetic dose has been shown to produce episodic memory impairments [4, 45]. Research seems to suggest that the memory for information received after drug administration is impaired, but not that received before drug administration, which means ketamine affects episodic memory principally by impairing the encoding of information into episodic memory rather than the recovery of previously encoded information from memory [46]. This property potentially blocks the effect of acute trauma on autobiographical memories, thereby reducing the development of trauma-related mental disorders without affecting the retrieval process of existing memories, indicating that ketamine can be used in acute trauma settings to prevent psychological complication. In all, we recommend ketamine as an ideal sedative agent for prehospital trauma care due to not only its excellent sedative and analgesic effects and safety but also its effect on neurocognitive process.

### 4.3. Role of Ketamine in Chronic Pain Management

Chronic pain is defined as pain lasting more than 3 months or longer than the expected time of tissue healing and usually results from central sensitization or neuropathic processes, with hyperalgesia or allodynia as common clinical manifestations [8]. Besides being used as an adjunct in the perioperative period, ketamine can be used to treat a variety of chronic pain syndromes. Intravenous injection of ketamine (1.4 mg/kg) daily for 10 days reduced various pain parameters in complex regional pain syndrome (CRPS) patients compared with saline [31]. For chronic trigeminal neuralgia, a single intravenous ketamine injection produced pain relief lasting up to 3 days, but only in patients who suffered pain for less than 3 years [36]. In addition, ketamine was reported to relieve chronic pain in patients with spinal cord injury [47, 48] and postherpetic neuralgia (PHN) [18, 49]. However, it is important to note that the sample size of these trials was small, resulting in this evidence being insufficient to support the long-term use of ketamine in chronic pain management.

Oral administration is also used although the poor bioavailability of oral ketamine presents many limitations [13]. A retrospective 5-year study showed that oral ketamine at a mean effective dose of 2 mg/kg/day produced pain relief in 44% of patients with chronic intractable pain, while about half of the patients reported adverse effects, mainly malaise, dizziness, or hallucinations [32]. In a recent RCT enrolling 42 patients with chronic neuropathic pain for over 6 months, researchers also showed that oral treatment of ketamine over 3 months significantly reduced VAS scores compared to baseline [33] although another RCT reported that oral ketamine for 16 days was equivalent to placebo for cancer-related neuropathic pain [50]. We speculate that the difference may be due to the difference in dosing duration. Due to a lack of data for oral administration of ketamine, we cannot conclude whether long-term use of ketamine for chronic pain is effective and safe right now. To confirm the availability of ketamine in chronic pain management, more well-designed and long-term RCTs are required in the future.

It is worth noting that the use of ketamine has been associated with the potential risk of liver and kidney function impairment. Research has shown that chronic use of ketamine can cause liver and kidney damage by inducing oxidative stress and inflammation, leading to tissue injury and dysfunction. Long-term use of ketamine has been shown to cause significant damage in liver, including fatty degeneration of liver cells, fibrosis, and increase in transaminase, and in kidney, including hydropic degeneration of the kidney tubules and atresia of glomeruli [51]. Also, the risk of ketamine-induced liver injury increases when the infusion is prolonged and/or repeated over a short period of time [52]. Moreover, even a single injection of ketamine can induce focal inflammatory cell infiltration in the livers of rats, although no changes are found in the kidneys [53]. Additionally, prolonged use of ketamine may result in ulcerative cystitis, characterized by symptoms such as increased urinary frequency, urgency, difficulty urinating, urge incontinence, and painful hematuria [4]. Therefore, caution should be exercised when using ketamine for chronic pain, particularly in patients with a history of liver or kidney disease. In these patients, the dosage and duration of ketamine use should be carefully monitored and adjusted as needed to avoid further harm.

In summary, ketamine seems to be effective in the treatment of a variety of chronic pain syndromes, while these indications are mainly based on some small or short-term trials and liver and kidney injuries from chronic use of ketamine are still considered a problem that needs to be solved. Further exploration will be needed to confirm the analgesic effect of ketamine in chronic pain management and to determine the underlying mechanism of action.

### 4.4. Role of Ketamine for Severe and Persistent Pain in Critically Ill Patients

Similar to the findings mentioned above, due to its ability to reduce the opioid requirements without suppressing hemodynamic parameters as seen with other sedative drugs, ketamine may also be considered an ideal adjunct analgesic and sedative agent for mechanically ventilated patients in the ICU setting. A retrospective study was conducted on mechanically ventilated patients who received adjunctive continuous infusion of low-dose ketamine and found a significant correlation between continuous infusion of ketamine and an increased reduction in opioid medication dosage, without adverse effects on hemodynamic stability [54]. Additionally, another retrospective cohort study observed that within 24 hours of initiating ketamine infusion, 63% of patients experienced a reduction or discontinuation of alternative sedative medications [55]. Recent work has also found that ketamine may reduce the promotion or the worsening of pain in critically ill COVID-19 patients [56]. However, while a retrospective review found an association between the initiation of ketamine and a decrease in the use of opioids and propofol, it has also been noted that this is correlated with an increase in the use of dexmedetomidine [57]. Considering the limited data on the use of ketamine as an adjunctive agent for analgosedation outside of procedural or general anesthesia, it is imperative to conduct larger, prospective, and randomized studies comparing ketamine with conventional sedation approaches.

## 5. Analgesic Mechanism of Action of Ketamine

### 5.1. Interactions with Multiple Binding Sites

The analgesic mechanism of action of ketamine is complex. The antagonism of NMDARs may be responsible for the specific properties of ketamine such as anesthesia, analgesia, amnesic, and psychosensory. Ketamine also interacts with other receptors, such as opioid and *γ*-aminobutyric acid (GABA) receptors.

**Figure 1:**
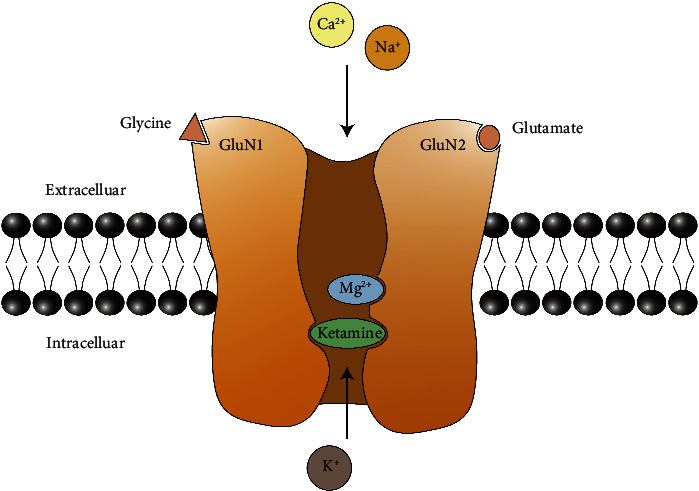
Molecular structure of NMDAR. The NMDAR is anchored in the cell membrane, and four subunits (commonly 2 GluN1 and 2 GluN2) form an NMDAR channel selective for the cations. The glycine binding site and glutamate binding site are located in the GluN1 and GluN2 subunit, respectively. When the membrane is not depolarized, even when two agonists occupy binding sites, the channel is blocked by the Mg^2+^ ion. Ketamine or other derivatives of phencyclidine can inactivate the receptor by binding to the intraductal PCP site.

The NMDAR is a kind of ionotropic glutamate receptor consisting of two GluN1 and two GluN2 subunits and has emerged as a common topic in several nervous system disorders, including ischemic brain injury, chronic neurodegenerative diseases, pain, depression, and schizophrenia [58, 59]. Due to the blockade by extracellular Mg^2+^ ions, the activation of NMDARs requires postsynaptic depolarization to release the Mg^2+^ ions besides binding of coagonists (glutamate and glycine), so-called voltage-ligand double-gated channel (shown in Figure 1) [58]. When the channel opens, calcium ions flow into the cell, which manifests as the activation of neurons. NMDARs are widely expressed in pain transduction pathways including the brain, spinal cord, and dorsal root ganglia [11], and there is substantial evidence that NMDAR activation is involved in the pain transmission procedure [11, 59, 60]. Ketamine, as an antagonist of NMDAR, can bind to an intrachannel site called the phencyclidine site so as to prevent the flow of ions (shown in Figure 1) [11]. The direct blockade of the pain transmission procedure is considered responsible for ketamine's analgesic effect. Local injection of ketamine could attenuate the pain induced by the mixture of kaolin and carrageenan in rats without effect on local edema, indicating that the relief of pain originated from the analgesic effect of ketamine [61]. In humans, the peripheral administration of ketamine could enhance the local actions of bupivacaine used for infiltration anesthesia and using ketamine alone could also induce a local anesthetic effect although lasting only 10–20 minutes [62]. In addition, because the binding site is located in the ion channel, the antagonism is more efficient if the channel has been previously in an open state, such as a chronic pain state [11]. The “use-dependent” mechanism may explain its analgesic properties in patients with chronic pain.

**Figure 2:**
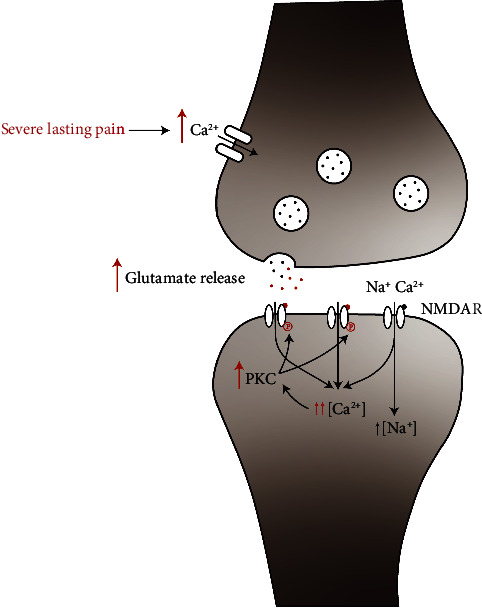
Schematic illustration of the role of NMDARs in central sensitization. In a physiological state, a noxious stimulus can be transmitted by glutamate and NMDARs. However, when suffering severe lasting pain (the red procedure), a constant drive of noxious afferent input leads to a flow of massive calcium ions into the presynaptic neurons, followed by increased release of glutamate into the synaptic cleft and more intense activation of NMDARs in postsynaptic neurons. More calcium entry causes activation of PKC and results in the phosphorylation of NMDARs. As a consequence, the magnesium block is decreased and channel opening time is prolonged.

Furthermore, researchers have found that the analgesic effect is not just due to the direct blockade of the pathway but also due to the inhibition of sensitization of the CNS. CNS sensitization, a product of increased dorsal horn neuron excitability, will result in two states: allodynia (a normal stimulus acting via low-threshold afferents then generates pain) and hyperalgesia (a noxious stimulus generates a pain response that is augmented in amplitude and duration) [60]. Convincing evidence has suggested that activation of NMDARs is involved in the development of spinal hyperexcitability and persistent pain [60]. Protein phosphorylation is a major mechanism for NMDARs to regulate the pain transmission procedure. In a state of pain, constant input of noxious signaling will lead to massive calcium ions flowing into the neurons, activating protein kinase C (PKC), which results in the phosphorylation of NMDARs. As a consequence, the Mg^2+^ block at resting membrane potentials is decreased and channels are easier to be opened (shown in Figure 2) [7, 63]. Besides phosphorylating NMDARs, PKC may modulate the NMDAR function by participating in their interactions with postsynaptic density and cytoskeletal proteins, especially postsynaptic density protein-95 (PSD-95) [60], which interacts with NMDARs and may implicate in the processing of spinal nociceptive information [7, 64, 65]. Ketamine can block the NMDARs and the aforementioned pathological changes, decreasing the amplification of the response to sensitization of the CNS [9].

However, the above mechanisms cannot explain why ketamine has an analgesic effect while some other antagonist of NMDARs, such as memantine, does not. We speculate that a phenomenon termed “trapping block,” which is associated with the off-rate of the compound, may explain the question. When glutamate has dissociated from its binding site on the NMDAR, a slow off-rate compound (ketamine) remains in the ion channel, like being trapped, thus causing a prolonged tonic blockade. In contrast, a fast off-rate antagonist (memantine) is capable to escape from the channel before it closes [5]. As a result, NMDARs can preserve more physiological function, hence preserving the pain transmission procedure.

The clinical effect of ketamine may not be entirely mediated by the NMDA receptor, and the opioid receptor system seems to partially mediate ketamine-induced analgesia. Although ketamine binds with very low affinity (28–100 *μ*M Ki values) at *μ*-, *δ*-, and *κ*-opioid receptors [11], the antinociceptive effect of ketamine was attenuated in *μ*-opioid receptor knockout mice compared to the wild-type mice [66]. In addition, researchers found that naloxone, a selective *μ*- and *δ*-opioid receptor antagonist, but not the *κ*-opioid receptor antagonist abolished ketamine-induced peripheral antinociception in a dose-dependent manner [67]. It is noteworthy that ketamine's antidepressant effect can alleviate pain-related negative emotions. In recent years, this effect has been considered to be involved in the analgesic process (as detailed in Section 5.3). Research has also found that naltrexone can attenuate the antidepressant effect of ketamine [68], offering an alternative explanation for why opioid receptor antagonists can block ketamine's analgesic effects. These findings indicate that opioid receptors participate in the analgesic effect of ketamine. However, the analgesic effects of ketamine in patients were not attenuated by naloxone [69], thereby discounting an opioidergic contribution to the analgesic effects evoked by ketamine. On the other hand, ketamine can exert an analgesic effect or enhance the effect of opioids by preventing the development of opioid-induced hyperalgesia [70, 71]. Opioid-induced hyperalgesia is the paradoxical increase in pain perception that often occurs in patients with chronic opioid treatment and consequently makes adequate pain treatment more difficult and sometimes even impossible [10]. The NMDAR antagonist can block and reverse hyperalgesia after the acute injection and continuous infusion of opioids [70, 71], which may explain why combining opioids with ketamine resulted in better analgesia and reduced consumption of opioids [23, 72].

Like other anesthetic agents, ketamine can potentiate *γ*-aminobutyric acid type A (GABAA) receptors to increase GABA inhibitory properties although only at high concentrations (more than 500 *μ*M) that are much higher than those obtained in the clinically relevant concentration range [73]. A recent study of neural circuits discovered that the activation of a distinct population of GABAergic neurons in the mouse central amygdala by general anesthetics (including ketamine) could suppress pain-related behaviors and abolish neuropathic pain-induced mechanical hypersensitivity [74]. We speculate that GABA, the most prevalent inhibiting neurotransmitter, seems to partly participate in the analgesia mechanism of action while further trials are needed to verify this.

### 5.2. Activation of Descending Inhibitory Pathway

The existence of endogenous mechanisms that diminish pain through descending pain modulatory pathways is generally accepted. The descending pathway mainly consists of the rostral anterior cingulate cortex (rACC), amygdala, midbrain periaqueductal gray region (PAG), rostral ventromedial medulla (RVM), locus coeruleus (LC), and spinal dorsal horns [75]. The RVM and LC are known to deliver descending serotoninergic and noradrenergic projections to the spinal cord to relieve the pain experience, respectively [76]. There is evidence that ketamine is able to influence descending pain inhibitory pathway. Ketamine can stimulate noradrenergic neurons and inhibit norepinephrine, dopamine, and serotonin uptake [1, 11]. Intraperitoneal injection of ketamine could significantly increase noradrenaline concentration in the medial prefrontal cortex of rats [77], and pretreatment with *α*2-adrenoceptor antagonist or serotonergic receptor antagonist completely reversed the analgesic effects of ketamine [78]. In addition, ketamine may be able to directly activate the descending inhibitory pathway and inhibit spinal dorsal horn nociceptive neurons. Regions of descending inhibitory pathway, such as ACC, insula, and brainstem, were found to be activated by low-dose ketamine in healthy volunteers by resting state functional MRI, and the changes were consistent with pain scores [79]. Low-dose ketamine could also enhance conditioned pain modulation (CPM), which was the central inhibition of a focal pain feeling by administering a second noxious stimulus in a remote area and was an important experimental expression of descending inhibition [80]. In conclusion, ketamine can activate the descending pain inhibitory pathway to relieve pain by strengthening the monoaminergic system or directly activating the corresponding brain regions.

### 5.3. Reduction of Pain-Related Negative Emotions

According to a multidimensional model proposed in 1986, chronic pain can be divided into three components including sensory-discriminative, affective-motivational, and cognitive-evaluative domains. In brief, the sensory-discriminative dimension is generated by input of nociceptive signals including information about pain location and magnitude; the affective-motivational dimension refers to the negative emotions that accompany pain and is processed at the level of the reticular and limbic structures; the cognitive-evaluative dimension provides contextual information and possible outcomes according to past experiences and is processed through higher CNS regions [9]. In recent years, an increasing number of research studies have been conducted on the antidepressant efficacy of ketamine, and the esketamine nasal spray was approved by FDA for treatment-resistant depression in 2019 [81]. Based on its antidepressant properties, research considers that its long-term benefits originate more from changes in the affective-motivational dimensions of pain, rather than just those in the sensory-discriminative dimension of pain [9]. In rodent chronic pain models, a single dose of ketamine can produce a persistent reduction in pain-related aversion, long after the termination of its analgesic effects in the sensory-discriminative dimension [82, 83]. The antiaversive property may be mediated by restoring functional connectivity among prelimbic-prefrontal cortex (PL-PFC) neurons [82] or suppressing the hyperactivity of neurons in the ACC, a brain region well known to modulate pain perception [83]. Consistent with the preclinical trial, a randomized, placebo-controlled trial that enrolled 19 subjects with CRPS types I and II in 2009 provided some similar evidence of ketamine's ability to modulate the sensory-discriminative and affective-motivational dimensions using the McGill Pain Questionnaire. All subjects received ketamine or saline infusions for 10 consecutive days. At week 12 after infusion, the average sensory-discriminative component was 31.1% lower than baseline while the affective-motivational component had decreased by 46.2% [31]. The results of these studies suggest that the long-lasting effects of ketamine on chronic pain could be attributable to changes in the affective-motivational component.

## 6. Metabolites of Ketamine

Ketamine is hepatically metabolized by several cytochrome enzymes [84]. Its metabolites include norketamine (80%), hydroxynorketamine (15%), and hydroxyketamine (5%) [85, 86]. Animal data show that norketamine is a nonselective NMDAR antagonist and has an analgesic effect on both acute and chronic pain, which is just 40% less compared to ketamine [87, 88]. In addition, the ketamine metabolite hydroxynorketamine is even superior to ketamine in reducing mechanical allodynia in acute and chronic pain models [89]. Unfortunately, there are no data on the contribution of its metabolites to analgesic effect in humans as these compounds are not available for human use. We can only infer the effect of these metabolites on analgesia by some indirect means. In a population pharmacokinetic-pharmacodynamic simulation model, it was estimated that norketamine contributed to the analgesic effects of a single 2 mg/kg ketamine bolus for 4 h [90]. Another study used a different design to assess norketamine's contribution. Subjects received a ketamine infusion after pretreatment with placebo or rifampicin. Rifampicin can competitively interact with multiple hepatic P450 enzymes involved in the N-demethylation of ketamine to norketamine. As the result of rifampicin pretreatment, the process of N-demethylation of ketamine is inhibited, leading to a much lower norketamine concentration in the plasma, while the ketamine concentration remains relatively unchanged. Surprisingly, pain relief was superior when the subjects had received rifampicin pretreatment, indicating a pronociceptive contribution of norketamine [91]. These results are contrary to previous animal experiments and other simulation clinical trials, but since this is not a direct demonstration of the analgesic effect of norketamine, further human studies may be required. Anyway, the metabolites of ketamine seem to contribute to the final analgesic effect of ketamine.

## 7. Conclusion

**Figure 3:**
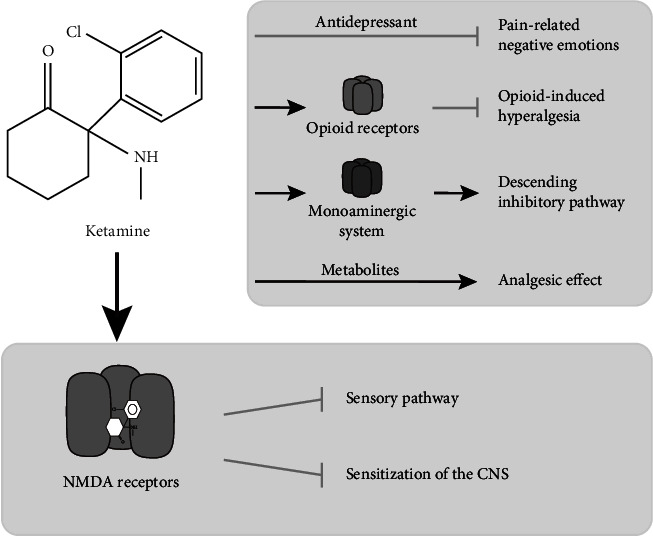
The possible mechanisms underlying NMDA analgesic properties. Ketamine primarily exerts its analgesic effects through the NMDA receptors (lower panel). The antidepressant effect, the opioid and monoaminergic system, and its metabolites seem to partly contribute to elicit analgesic properties (upper panel).

Having been in use for more than 50 years, ketamine has proven to be a safe anesthetic drug with potent analgesic properties and it can be used in a variety of surgical procedures and pain syndromes to improve pain therapy. The role of NMDAR in nociceptive transmission has been established in humans, and the noncompetitive binding of ketamine to the phencyclidine binding site of NMDARs may partly account for its analgesic property. Apart from its well-known NMDAR direct blockade, ketamine also inhibits the sensitization of the CNS, which allows a reduction of allodynia and hyperalgesia. Besides its interaction with NMDAR, ketamine may potentially interact with a wide range of receptors and modulation systems involved in the pain transmission pathway. With increasing research on the antidepressant effects of ketamine, there is a growing consideration that ketamine may participate in the analgesic effects by alleviating pain-related aversive emotions. Moreover, the metabolite of ketamine has also been found to have analgesic effects, which can prolong pain relief. Taken together, ketamine's analgesic mechanism of action appears to be a complex combination of multiple factors including those mentioned above (shown in Figure 3). Further exploration is needed to confirm the indications for ketamine analgesia and to further elucidate its analgesic mechanism.

## Figures and Tables

**Table 1:** Summary of parts of representative clinical trials regarding various possible actions of ketamine described in this review.

Type of study	No. of patients	Type of patients	Treatment of ketamine	Effect
*As an adjunct to general anesthesia*				
Double-blind randomized, placebo-controlled clinical trial	50	Patients undergoing elective arthroscopic meniscal surgery	0.15 mg/kg i.v. after anesthetic induction	Adding small-dose ketamine improved postoperative analgesia after knee arthroscopy [24]
Double-blind randomized, placebo-controlled clinical trial	121	Patients undergoing elective distal or total gastrectomy	A bolus dose of 1.0 mg/kg i.v., maintained at 0.5 mg/kg·h until skin closure	With intravenous ketamine, visual analog scale scores and morphine consumption were significantly lower [25]
Double-blind randomized, placebo-controlled clinical trial	75	Patients undergoing elective unilateral total knee replacement	A bolus dose of 0.2 mg/kg i.v. ketamine, followed by a 0.12 mg/kg·h continuous infusion until the end of surgery and 0.06 mg/kg·h until the second postoperative day	Ketamine produces opioid-sparing, decreases pain intensity, and improves mobilization after total knee replacement [26]
Double-blind randomized, placebo-controlled clinical trial	202	Patients undergoing posterior cervical or lumbar spinal surgery	A bolus dose of 1 mg/kg i.v. ketamine, followed by continuous ketamine 0.042/0.083 mg/kg·h for 24 h	Pain scores and analgesia requirement in the 0.083 mg/kg·h group were significantly lower [27]

*As an adjunct to peripheral or intravertebral anesthesia*				
Double-blind randomized, placebo-controlled clinical trial	37	Patients undergoing unilateral knee arthroplasty	Ropivacaine (10 mg/ml, 10–20 ml), with esketamine 0.25 mg/kg, lumbar epidural anesthesia	The combination of esketamine and ropivacaine in epidural anesthesia increased postoperative pain relief [28]
Double-blind randomized, clinical trial	60	Children (6 months to 10 years) undergoing inguinal herniotomy	0.75 ml/kg of bupivacaine 0.25% with ketamine 0.25/0.5/1.0 mg/kg, caudal epidural block	The addition of 0.5 mg/kg ketamine to caudal epidural block effectively enhanced postoperative pain relief without increasing side effects [29]
Double-blind randomized, clinical trial	50	Children undergoing inguinal herniotomy	0.5 mg/kg ketamine, with or without 1.0 ml/kg of bupivacaine 0.25%, caudal epidural block	The bupivacaine-ketamine mixture provided better analgesia than the bupivacaine solution alone [30]

*Chronic pain*				
Double-blind randomized, placebo-controlled clinical trial	19	CRPS patients	For 10 days, a daily intravenous infusion of 100 mg of ketamine is administered, with each infusion lasting for 4 h per day	Intravenous ketamine administered in an outpatient setting resulted in significant reduction in many pain parameters [31]
Retrospective 5-year study	51	Patients with chronic pain syndromes, unresponsive to usual treatment for several years	① Intravenous in-hospital administration, then a conversion to an oral route ② Oral treatment directly administered at home	Pain was reduced or abolished in two-thirds of patients under ketamine therapy; ketamine was effective for patients taking opioids and resulted in few adverse effects [32]
Double-blind randomized, active-controlled clinical trial	42	Patients who had experienced neuropathic pain for more than 6 months	3 mg ketamine, oral treatment	Oral ketamine is effective in treating chronic neuropathic pain [33]

## Data Availability

The data used to support the findings of this study are available from the corresponding author on reasonable request.
